# Weber C ankle fractures with tibiofibular diastasis: syndesmosis-only fixation

**DOI:** 10.1590/1413-785220172503151204

**Published:** 2017

**Authors:** Serkan Sipahioglu, Sinan Zehir, Erdem Isikan

**Affiliations:** 1 Harran University Medical Faculty, Department of Orthopedics and Traumatology, Sanliurfa, Turkey; 2 Hitit University Medical Faculty, Department of Orthopedics and Traumatology, Corum, Turkey

**Keywords:** Ankle injuries, Fracture fixation, interna, Fractures, bone, Follow-up studies

## Abstract

**OBJECTIVES:**

To evaluate syndesmosis-only fixation in Weber C ankle fractures with tibiofibular diastasis and to assess the need for additional fibular fixation.

**METHODS:**

Twenty-one patients with Weber C ankle fractures and tibiofibular diastasis were followed for at least 24 months after treatment. In treatment of the Weber C fractures, only a syndesmosis screw was used through a mini open lateral incision if the syndesmosis could be anatomically reduced and fibular length and rotation could be restored. At follow-up, anteroposterior tibiofibular distance, lateral fibular distance, medial mortise distance and fracture healing were compared and patients were clinically evaluated using the Olerud and Molander ankle scale scoring system.

**RESULTS:**

The average duration of follow-up was 49 months and the decreases in anteroposterior tibiofibular distance and lateral fibular distance were statistically significant. At the last follow-up the average clinical score was 86. Ankle mortise was reduced at follow-up in all cases except one, which resulted in a late diastasis.

**CONCLUSIONS:**

Syndesmosis-only fixation can be an effective method of treating Weber type-C lateral malleolar fractures with syndesmosis disruption in cases where intraoperative fibular length can be restored and anatomical syndesmosis reduction can be achieved. ***Level of Evidence IV, Case Series.***

## RESUMO

**OBJETIVOS:**

Avaliar a fixação apenas da sindesmose em fraturas do tornozelo de Weber tipo C com diástase tibiofibular e a necessidade de fixação adicional.

**MÉTODOS:**

Vinte e um pacientes com fraturas de tornozelo Weber C e diástase tibiofibular foram seguidos por pelo menos 24 meses após o tratamento. No tratamento dessas fraturas, apenas um parafuso para sindesmose foi colocado através de mini-incisão lateral e se a sindesmose pudesse ser anatomicamente reduzida e o comprimento e a rotação da fíbula pudessem ser restaurados. No seguimento, a distância tibiofibular anteroposterior, a distância fibular lateral e a distância medial do encaixe do tornozelo e a consolidação das fraturas foram comparados e os pacientes foram avaliados clinicamente pelo sistema de pontuação da escala de tornozelo de Olerud e Molander.

**RESULTADOS:**

A duração média do seguimento foi de 49 meses e as diminuições da distância tibiofibular anteroposterior e fibular lateral foram estatisticamente significantes. No último seguimento, a pontuação clínica média foi de 86. O encaixe do tornozelo foi reduzido em todos os casos, exceto um, que resultou em uma diástase tardia.

**CONCLUSÕES:**

A fixação apenas da sindesmose pode ser um método eficaz de tratamento de fraturas laterais Weber tipo C com lesão na sindesmose, nos casos em que o comprimento fibular intraoperatório pode ser restaurado e a redução anatômica da sindesmose possa ser obtida. Nível de Evidência IV, Série de Casos.

## INTRODUCTION

Syndesmosis injuries occur in 10% of ankle fractures and approximately 20% of ankle fractures that require internal fixation, with an incidence of approximately 15 per 100,000 in the general population. Syndesmotic injuries are most commonly caused by pronation-external rotation, pronation-abduction and less frequently supination external rotation mechanisms. As the talus abducts or rotates externally in the mortise, one or more syndesmotic ligament disruptions can occur. Initial rupture of the deltoid ligament or fracture of the medial malleolus consequently occurs and if the trauma is severe enough may be followed by rupture of the anterior tibiofibular ligament and interosseous membrane. These events may be at the syndesmotic level (Danis-Weber type B injury) or the supra-syndesmotic level (Danis-Weber type C injury); both are associated with fracture of the fibula. ^(^ ^1^

To avoid diastasis of the ankle joint, an injured distal tibiofibular syndesmosis should be reduced.^1^ Osteoarthritis and poor functional outcomes are more common in patients with a widened mortise.^2^ Although numerous methods have described the conventional procedure, stabilization with a syndesmotic screw remains the most popular treatment. It is not possible to consistently estimate the integrity of the interosseous membrane and subsequent need for trans-syndesmotic fixation based solely on the level of the fibular fracture and an intraoperative syndesmotic stress test is consequently recommended to establish the presence or absence of syndesmotic instability. This has led to controversy over decisions related to syndesmotic fixation based on the level of the fibular fracture. Traditionally, a diastasis screw is recommended if the fibular fracture is more than 3.5 cm above the top of the syndesmosis and the deltoid ligament is injured. In cases with a medial malleolar fracture, after the fracture has been rigidly fixed a diastasis screw is used if the fibular fracture is more than 15 cm above the syndesmosis.^3^

Since the indications for syndesmotic screw application are clear, questions arise about the role of internal fixation of the fibula in an associated fibula fracture after syndesmosis reduction. When the fibular fracture is located in the middle or proximal one-third of the diaphysis, problems with performing an additional procedure may arise in cases where soft tissue is compromised. If the syndesmosis can be anatomically reduced and stable fixation is achievable, internal fixation of the fibula may not be required. This study presents the results of syndesmosis-only fixation in patients with Weber C ankle fractures with tibiofibular diastasis.

## MATERIALS AND METHODS

Twenty-three patients with supra-syndesmotic fibular fractures whose distal tibiofibular syndesmotic injuries were fixed by syndesmosis alone were studied retrospectively. Patients with multiple injuries, late diagnosis and open fractures were not included. All patients underwent ankle X-rays that included bilateral anteroposterior (AP), AP 15-degree internal rotation (mortise) and lateral views for comparison and to determine whether there was a tibiofibular diastasis. Syndesmosis was determined by measuring the horizontal distance between the medial cortical border of the fibula and the radio dense line of the tibiofibular notch 1 cm above the ankle joint. We also evaluated the anteroposterior X-rays to assess the distance between the medial malleolar lateral articular side and medial talus side, designated as the medial mortise distance (medial clear space). On the lateral radiograms, the distance measured between the fractured ends of the fibula was recorded as the lateral fibular distance. Patients with a tibia-fibular clear space exceeding five millimeters were diagnosed with tibiofibular diastasis and treated with syndesmosis fixation. 

Patients underwent surgery when the soft-tissue swelling decreased. Syndesmotic disruption was confirmed under fluoroscopy using the external rotation stress test; the proximal tibia was stabilized using the examiner's or an assistant's hand, the examiner held the ankle in neutral flexion at a 90 angle between the tibia and foot and consistent external rotational force was applied to the ankle mortise. The external rotation stress test was positive in all patients. Proximal fibula fractures with syndesmosis injury (Maisonneuve fracture) were excluded from the study since fibula fixation is unnecessary for these fractures.^4^

**Figure 1. (A:**
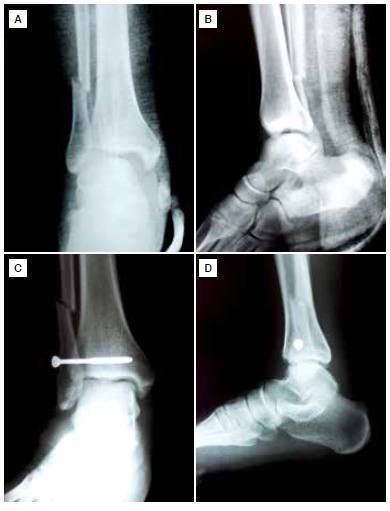
and B) Weber C ankle fracture with tibiofibular diastasis, (C and D). Postoperative radiograph with syndesmosis-only fixation.

During surgery, the medial ankle structures were internally fixed first when necessary. A malleolar screw was used for medial malleolar fixation in all patients. In cases where it was possible to anatomically reduce the syndesmosis and restore fibular length, a syndesmosis screw was placed through a mini open lateral incision. Restoration of fibular length and rotation was decided using fluoroscopy and Shenton's line of the ankle was assessed. A malleolar screw was used for the tibiofibular diastasis as a syndesmotic screw; 4.0 mm stainless steel malleolar screws were used in all cases and tricortical fixation was performed using a single screw. The diastasis screws were placed parallel and 2 to 3 cm above the ankle joint, 20 to 30 degrees antero-medially after clamp stabilization of the syndesmosis was achieved. A tourniquet was used and antibiotic prophylaxis was initiated prior to surgery. A short leg plaster splint was used postoperatively in all patients. (Figures 1A-D) Non-weight-bearing mobilization using crutches was recommended for six weeks. At six weeks, the ankle was X-rayed to assess whether the ankle mortise was well-reduced and stable and if the fracture was healing. If no problems were seen, the diastasis screws were removed under local anesthesia six to eight weeks after the surgery. Ankle motion and partial weight bearing were subsequently permitted. 

At follow-up, we compared X-rays including measurements of the anteroposterior tibiofibular distance, lateral fibular distance and medial mortise distance (including preoperative, postoperative and final control films). During the last follow-up visit, the patients were clinically evaluated using the Olerud and Molander^5^ ankle scale (OMAS). The OMAS is a self-administered objective scoring system patients complete using a questionnaire. In this functional rating scale, the score ranges from 0 (totally impaired) to 100 (completely unimpaired). Scores are based on nine different items: pain, stiffness, swelling, stair climbing, running, jumping, squatting, supports and activities of daily living. Scores of 91-100 were graded as excellent, 61-90 as good, 31-60 as fair and 0-30 as poor.

Statistical analysis was performed using SPSS 12.0 software (SPSS Inc., Chicago, IL, USA). Preoperative and postoperative differences were compared using the Wilcoxon signed rank test. The significance level was set at *p* <0.05. All subjects gave informed consent to participate in the study and the protocol was approved by the institutional review board under reference number 125/2009.

## RESULTS 

The average patient age was 40.1 ± 11.7 years (range 20-61). Thirty percent (n= 7) of the patients were women. The mechanism of injury was a twisting sprain in 15 patients, sports-related in five patients and traffic accident in three patients. All of the fractures were in the middle third of the fibula. Seventeen fractures were pronation-external rotation (PER) injuries according to the Lauge-Hansen classification (stage 3 PER in 13 ankles and stage 4 PER in four ankles) and six were pronation-abduction type. Medial malleolar screw fixation was performed in 12 ankles and a deltoid ligament repair was performed in one case. There were no soft tissue infections. On the control examinations, delayed union was observed in two patients. Complex regional pain syndrome (CRPS) developed in one patient and he was referred to the physiotherapy department. 

The average duration of follow-up was 48.3 ± 19.1 months (range 22-78 months). The average anteroposterior tibiofibular distance was 6.8 ± 2.5 mm (range 14-2 mm) preoperatively and 3.4 ± 1.0 mm (range 6-2) postoperatively. The average lateral fibular distance was 2.0 ± 1.2 mm preoperatively and 1.2 ± 0.8 mm postoperatively. The decrease in anteroposterior tibiofibular distance and lateral fibular distance was statistically significant. The average medial mortise distance was 3 ± 1.9 mm (range 2-11 mm) preoperatively and 2.1 ± 0.5 mm (range 1-3 mm) postoperatively. The change in the medial mortise distance was not statistically significant. When the last control radiographic measurements were compared with the postoperative measurements, there was no statistically significant change in their values. 

At the last follow-up, the average score according to the OMAS scoring system was 85.8 ± 8.2 (range 64-94). Five patients (22%) had an excellent outcome, 16 patients (69%) had a good outcome and two patients (9%) had a fair outcome. The ankle mortise was reduced in all cases except one, which resulted in a late diastasis; revision surgery with bone grafting and internal fixation of the fibula was performed in this patient. One patient had residual ankle stiffness which responded to intensive physiotherapy. None of the syndesmotic screws broke.

## DISCUSSION

The most important predictor of a good functional outcome for ankle fractures with a syndesmotic injury is the anatomical reduction of the syndesmosis.^2^ Displacement greater than 1 mm of the talus on the mortise X-rays in comparison with the contralateral extremity is accepted as an indication for surgery. Restoration of fibular length and achieving correct rotation of the fibula are essential for restoring the proper tibiofibular relationship. A residual lateral displacement of the talu (exceeding 2 mm) is associated with a 49% increase in articular mean pressure and a greater than 90% chance of degenerative changes and poor outcomes. The distal fibular fragment has been shown to rotate externally relative to the proximal fragment, so internal rotation of the distal fragment is essential to achieve reduction.^6^

Lateral plating of the fibula in proximal and diaphysis fractures involves additional soft tissue dissection, which is associated with a risk for injury to the common peroneal nerve and anatomical plate incongruity. Though overall complication rates are low and outcomes are usually good, soft tissue problems are reported in up to 22% of cases. Patients with a compromised soft tissue envelope resulting from either the injury or other co-morbidities including diabetes mellitus peripheral vascular disease are prone to high complication rates.^7^ To avoid compromising the thin lateral soft tissues, a dorsal approach with application of an "antiglide" plate to the posterior aspect of the fibula has been described.^8^ Some reports also describe superficial peroneal nerve variations in anatomic study with the possibility of injury to the intermediate dorsal cutaneous nerve.^9^ Kim et al.^10^ suggested anterior transposition of this nerve to reduce the incidence of symptoms related to superficial peroneal nerve injury after fixation of the lateral malleolus. Redfern et al.^11^ found significantly more symptoms associated with the superficial peroneal nerve in a surgically-treated group (21%) compared to patients who were treated conservatively (9%) 2 years after an ankle fracture. In order to avoid these problems, indirect fixation of the fibula is possible using syndesmosis fixation after the correct length and rotation of the fibula and reduction of the syndesmosis is achieved. Syndesmosis fixation alone was performed according to the level of the fibular fracture, preoperatively and only high-level fractures were selected. The surgical procedure was performed to restore fibular length and reduce the syndesmosis. If these conditions could not be obtained preoperatively or intraoperatively, additional fixations were performed. 

Some investigators suggest that there are no optimal radiological parameters to assess the integrity of the syndesmosis.^12^ The Hook test in the sagittal plane is considered a sensitive assessment of instability. In this study, the Hook test was used for diagnosis in addition to radiological assessment of the syndesmosis. The medial clear space, tibiofibular overlap and tibiofibular clear space should be accurately restored in the mortise view and the Shenton line of the ankle should be unbroken. Lateral imaging of the ankle is useful, together with comparison views of the normal ankle so that the required result can be achieved. The anteroposterior tibiofibular distance, lateral fibular distance and medial mortise distance were used to evaluate the reduction. Only a decrease in the medial mortise distance was not statistically significant. Because the medial malleolus was also fractured in 12 patients, the change in the medial mortise distance was not statistically significant.

In a cadaver study, Ho et al.^13^ compared syndesmotic fixation alone and syndesmotic fixation with the addition of a fibular plate to determine whether the addition of a fibular plate would lead to better biomechanical properties. The results showed higher rotational stability, load to failure and stiffness with the plate compared to the syndesmotic fixation-only technique. In addition, a syndesmosis study in cadavers with stable fixation of bimalleolar fractures showed that if the fibular fracture was within 4.5 centimeters of the joint, syndesmotic fixation was not necessary.^3^ However, one clinical study found a high failure rate when PE-4 fractures with deltoid ligament disruption and fibular fractures distal to this "critical zone" were treated with fibular fixation only.^14^ The investigators concluded that trans-syndesmotic fixation is indicated in all PE-4 fractures with rupture of the deltoid, regardless of the location of the fibular fracture. In the case of a supra-syndesmotic stage 4 PER injury, reported by Saltzman, syndesmotic fixation alone restored the length and rotation of the distal fibular segment, thereby achieving a congruent ankle mortise,^15^ which provided a buttress against lateral talar subluxation. Diaphyseal fibular fractures, in which the syndesmosis and ankle joints are congruent, are treated non-operatively with high rates of union and low complication rates. Internal fixation of such diaphyseal fibular fractures is associated with risks (e.g. infection, neurological damage, prominent metal work, peroneal tendonitis, nonunion and delayed union and hardware failure) that outweigh the benefits of such procedures. Mohammed et al.^16^ performed syndesmosis fixation alone in 12 patients with Weber-C ankle fractures with syndesmotic injury and reported good to excellent outcomes in 83% of these cases. As a result, syndesmosis-only fixation was recommended as an effective treatment option for the combination of syndesmosis disruption and Weber type-C lateral malleolar fractures.

Removal of the fixation screw is recommended at week eight or nine.^6^ ^,^ ^17^ Burgert and Jones^18^ reported that six weeks was not sufficient. Some investigators have reported that early mobilization and weight bearing should be encouraged.^19^ Some suggest that the screw should not be removed and that any additional surgery in an incompletely healed wound may cause higher rates of infection.^20^ Ebraheim et al.^17^ suggested that the syndesmotic screw should not be removed until the fibular fracture shows signs of healing, especially in cases with a deltoid ligament injury. In an internal fixation series containing 32 patients with supra-syndesmotic ankle fractures, these authors found a 6% rate of delayed union and 13% rate of nonunion, as well as one late diastasis of the syndesmosis following screw removal after six weeks; as a result, fibular fracture healing should be assessed prior to screw removal. 

Syndesmosis-only fixation can be an effective method for treating patients with Weber type-C lateral malleolar fractures with syndesmosis disruption. However, restoration of fibular length and anatomical reduction of the syndesmosis are essential for a successful outcome. Fibular fracture healing should be assessed prior to removal of the diastasis screw at the appropriate time. We recommend fixing syndesmosis with only a screw after anatomic reduction if fibular length is restored in Weber C fractures.

## CONCLUSIONS

Syndesmosis-only fixation can be an effective method of treating patients with Weber type-C lateral malleolar fractures with syndesmosis disruption in cases where intraoperative fibular length restoration and anatomical syndesmosis reduction can be achieved.
